# The role of ants in north temperate grasslands: a review

**DOI:** 10.1007/s00442-017-4007-0

**Published:** 2017-11-16

**Authors:** B. D. Wills, D. A. Landis

**Affiliations:** Department of Entomology and DOE Great Lakes Bioenergy Research Center, Michigan State University, East Lansing, MI USA

**Keywords:** Ecosystem services, Plant–insect interactions, Biodiversity, Beneficial insects, Temperate

## Abstract

Historic and current land-use changes have altered the landscape for grassland biota, with over 90% of grasslands and savannas converted to agriculture or some other use in north temperate regions. Reintegrating grasslands into agricultural landscapes can increase biodiversity while also providing valuable ecosystem services. In contrast to their well-known importance in tropical and subtropical ecosystems, the role of ants in temperate grasslands is often underappreciated. As consumers and ecosystem engineers, ants in temperate grasslands influence invertebrate, plant, and soil microbial diversity and potentially alter grassland productivity. As common and numerically dominant invertebrates in grasslands, ants can also serve as important indicator species to monitor conservation and management practices. Drawing on examples largely from mesic, north temperate studies, and from other temperate regions where necessary, we review the roles of ants as consumers and ecosystem engineers in grasslands. We also identify five avenues for future research to improve our understanding of the roles of ants in grasslands. This includes identifying how grassland fragmentation may influence ant community assembly, quantifying how ant communities impact ecosystem functions and soil processes, and understanding how ant communities and their associated interactions are impacted by climate change. In synthesizing the role of ants in temperate grasslands and identifying knowledge gaps, we hope this and future work will help inform how land managers maximize grassland conservation value while increasing multiple ecosystem services and minimizing disservices.

## Introduction

Grassland ecosystems support high levels of plant and animal biodiversity, but are increasingly threatened by global change drivers including land-use change, climate change and invasive species. Temperate grasslands and savannas have been particularly impacted and currently represent the single most highly converted and least protected biome globally (Hoekstra et al. 2005; Orlikowska et al. 2016). For example, in North America past conversion of grasslands to agriculture and other uses has resulted in the loss of more than 90% of the former total area of mixed grass prairies (Samson and Knopf 1994) and conversion continues to occur. Recent interest in corn ethanol production in North America has contributed to the continued loss and fragmentation of North American grasslands (Wright and Wimberly 2013). Similar rates of grassland loss have also occurred throughout Europe (Ratcliffe 1984; Poschlod and WallisDeVries 2002) and over vast portions of East Asia (Tsukada et al. 2004). In view of these losses and global vulnerability (Hoekstra et al. 2005), there is great interest in grassland conservation and developing creative ways to reintegrate grasslands into agricultural landscapes (Williams et al. 2013; Liebman and Schulte 2015). Such managed grasslands can increase biodiversity, productivity, and the provision of ecosystem services from agricultural landscapes (Werling et al. 2014; Landis et al. 2017), but require a deep understanding of the roles played by ecologically dominant taxa.

Ants (Hymenoptera: Formicidae) are common, dominant taxa in terrestrial ecosystems that play key roles in shaping ecosystem structure and function. However, our understanding of the role of ants in temperate grasslands remains incomplete. In grasslands globally—including tropical and subtropical regions—ants are known to play important roles as consumers and ecosystems engineers (Hölldobler and Wilson 1990; Del Toro et al. 2012), that influence invertebrate, plant, and soil microbial diversity (Boulton and Amberman 2006; Sanders and van Veen 2011), and have the potential to alter grassland productivity (Dean et al. 1997). As dominant and influential members of grasslands, ants can serve as indicator species to monitor conservation and management practices (Underwood and Fisher 2006; Moranz et al. 2013). Far less is known about the role of ants in temperate grasslands, particularly their role in pest suppression, soil nutrient cycling, microbial community, and plant community regulation (Frouz et al. 2003; Nemec 2014).

Here, we review the role of ants in north temperate grasslands, with a focus on the implications for biodiversity conservation and the provision of ecosystem services. We provide an overview of their ecological roles, highlight their individual interactions, and identify knowledge gaps in our understanding of the role ants play in the regulation of grassland productivity and biodiversity. Where necessary we will use examples from other temperate systems to discuss importance of ants as consumers and ecosystem engineers. We then identify five avenues for future research to improve our understanding of the roles of ants in north temperate grasslands. By synthesizing current understanding and identifying knowledge gaps, we hope to improve data available for land managers to maximize conservation value while increasing multiple ecosystem services and minimizing disservices.

## Overview of the role of ants in grasslands

**Fig. 1:**
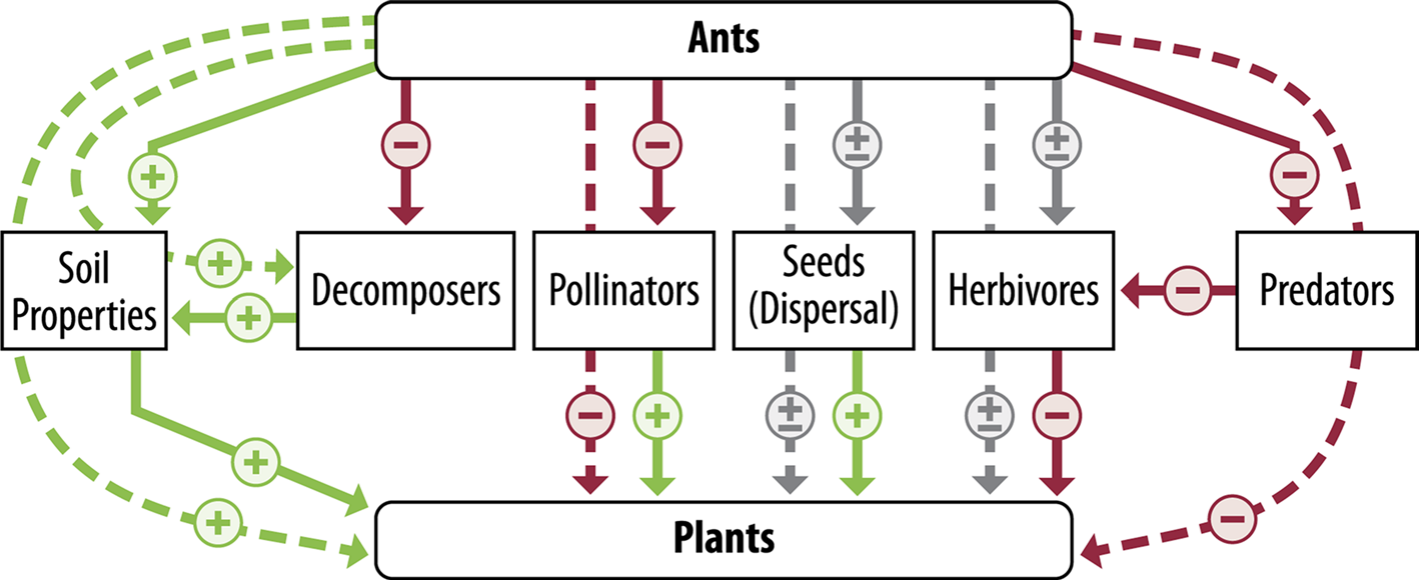
Diagram of the interaction between ants, soil properties, and other organisms. Lines represent the direct (solid) and indirect (dashed) impacts on organisms. Red lines represent negative interactions, green lines represent positive interactions and gray represents an interaction that can be either positive or negative depending on the species considered. Ants generally have a positive effect on soil properties through nest construction and maintenance, improving soil conditions for plants. Altered soil conditions can also improve conditions for decomposers but ants can directly diminish decomposer abundance as consumers. By improving soil conditions for microbial decomposers, ants improve soil conditions for plants. Ant forging can negatively impact predator and pollinators through direct or indirect interactions. These can negatively impact pollination success or reduce beneficial predators. Seed collecting or seed harvesting ants can serve as either seed predators (− effect) or seed dispersers (+ effect). Ants can protect honeydew-producing insects, negatively impacting plants. In tending honeydew-producing insects, ants can disrupt herbivores and reduce plant herbivory. When honeydew-producing insects are present, the overall indirect effect of ants on plants is positive

**Fig. 2:**
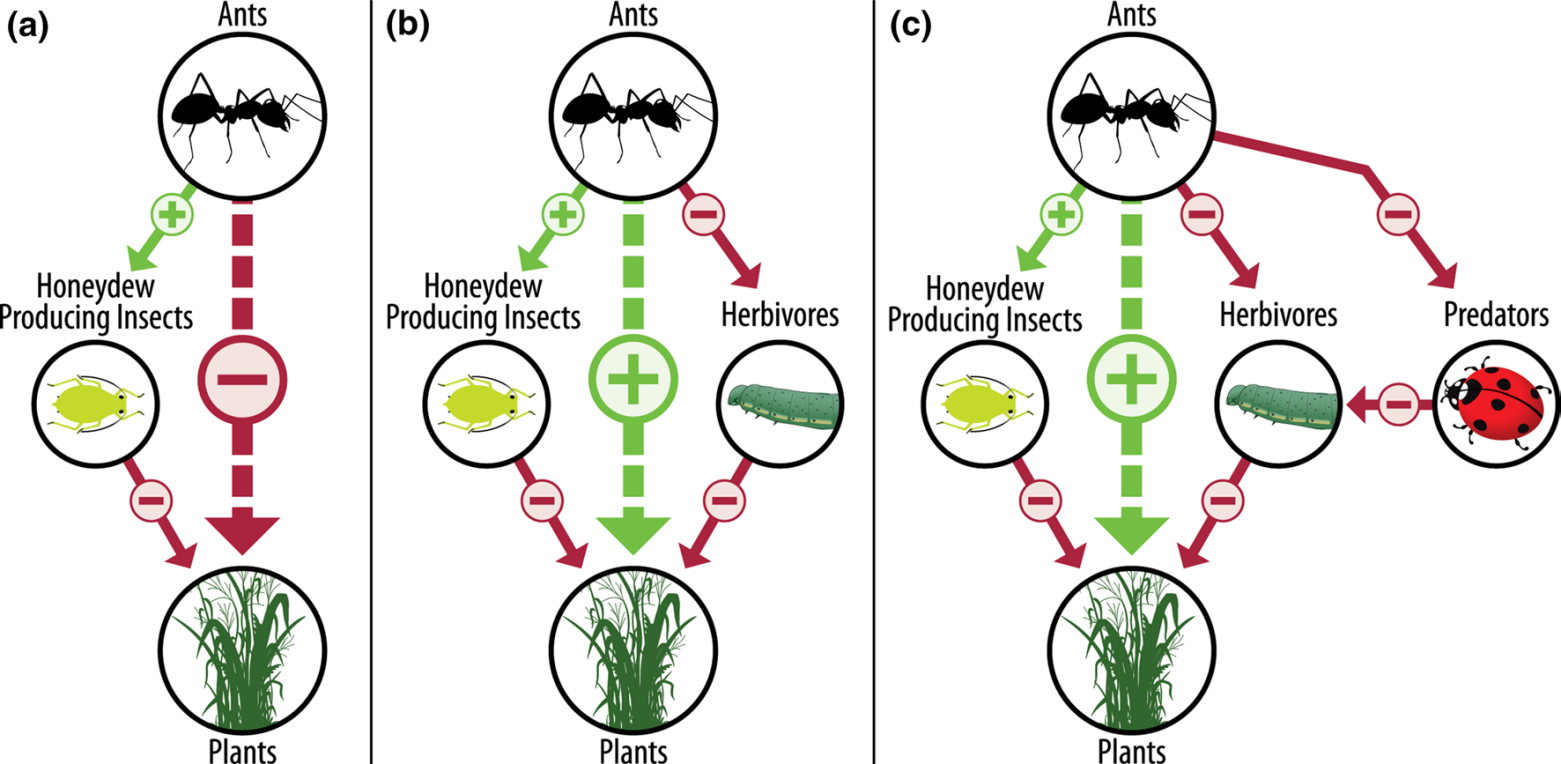
The direct (solid) and indirect (dashed) effects of ants on plants. **a** With access to honeydew-producing insects (e.g., aphids), ants can indirectly have a negative impact on plants by protecting and dispersing plant pests. **b** With access to honeydew-producing insects and herbivores present, ants in this system indirectly benefit plants because ant foraging for honeydew reduces herbivory. **c** If honeydew-producing insects and both herbivores and predators present, ants can negatively impact populations of predators and herbivores in protecting honeydew resources. Despite reducing other predators, in negatively impacting herbivore feeding and abundance, the overall indirect effect of ants is positive

The interactions between ants and other organisms is well supported (Rosumek et al. 2009; Del Toro et al. 2012), but can be complex. As consumers, ants directly and indirectly impact abundance, diversity, and behavior of other arthropods within an ecosystem (Fig. 1). Ants can directly reduce populations of other organisms (herbivores, predators, etc.) through predation or competition (Styrsky and Eubanks 2007; Sanders et al. 2011). They can also indirectly influence arthropod populations through non-consumptive effects, where cues to the predator’s presence (e.g., visual, chemical) cause changes in the development, growth, or behavior of potential prey (Cembrowski et al. 2014; Mestre et al. 2014). The nature of these interactions (positive or negative/direct or indirect) towards other arthropods generally depends on access to plant-based carbohydrate resources (e.g., extrafloral nectar, hemipteran exudates). The defense of plant-based carbohydrate resources determines the likelihood of ants protecting honeydew-producing plant pests (Kaplan and Eubanks 2005), or reducing the presence of herbivores (Pringle et al. 2011), predators (Sanders and Platner 2007), or pollinators (Galen and Geib 2007) (Fig. 2). While in other cases, ants are known to more directly influence other arthropod populations and alter trophic food webs regardless of access to carbohydrate resources (Sanders and Platner 2007).

Ant forging also influences their roles as ecosystem engineers. As seed dispersers, ants can redistribute seeds within a landscape and help maintain a mosaic of plant diversity (King 1977b; Dean et al. 1997). Their foraging activity can also concentrate resources within a nest and influence the availability of resources within the soil by increasing microbial activity, and the subsequent release of nutrients (Lobry de Bruyn and Conacher 1990; Jouquet et al. 2006). In addition, nest construction and maintenance behaviors can influence water infiltration and retention by increasing biopores for water to move through the soil (Lobry de Bruyn 1999; Cammeraat et al. 2002). Nest construction and maintenance can also redistribute soil, organic matter, and nutrients within the soil profile (Lobry de Bruyn 1999; Halfen and Hasiotis 2010). By both disturbing and redistributing soil and seeds within a landscape, ants can have a dramatic impact on grassland diversity and plant structure (Dean et al. 1997; Dostál 2007).

Ants frequently play an important role in determining grassland biodiversity. The impact of ants on other invertebrates influences their distribution, abundance, and diversity within a grassland (Boulton and Amberman 2006; LeBrun et al. 2013). The direct effects on these groups can indirectly alter plant productivity and biodiversity (Dean et al. 1997; Dostál 2007). Although these indirect interactions are complex and may work in opposing directions, recent studies exploring the complexity of these interactions suggests the overall effect of ants on plants is positive (Styrsky and Eubanks 2007; Rosumek et al. 2009) (Figs. 1, 2). Moreover, ant forging can help maintain plant diversity by dispersing seeds or creating conditions for plant colonization (Dostál 2007). By better understanding ants as important components in grasslands, we hope to improve grassland management and conservation.

## Ants as consumers in grasslands

In the following section, we review the role of ants in north temperate grasslands, the interactions between ants and other actors (Table 1), and then ultimately discuss how these interactions affect the distribution, abundance, and productivity of grassland plant species.

**Table 1:** Examples of ant species from north temperate grasslands and their associated effects as consumers and ecosystem engineers

Ant species	Location	Effects	References
Ants as consumers			
*Aphenogaster rudis*	USA	Keystone seed disperser	Pudlo et al. (1980) and Ness et al. (2009)
*Formica clara*	Germany	↑Predator dispersal via chemical cues	Mestre et al. (2014)
*Formica cunicularia*	Germany	↓Spiders and lepidoptera density	Sanders and Platner (2007)
*Formica exsecta*	Switzerland	More seeds and ↑species richness on mounds	Schütz et al. (2008)
*Formica japonica*	Japan	Tends aphids, ↓lepidoptera density, and ↓leafhopper density	Ando and Ohgushi (2008)
*Formica* spp.	USA	Tend membracids and ↓leaf beetles (*Trirhabda virgata* and *T. borealis*) herbivory	Messina (1981)
*Formica subsericea*	USA	Seed disperser	Pudlo et al. (1980)
*Lasius niger*	Germany	↑Herbivores, generalist predators, and parasites	Sanders and van Veen (2011)
*Lasius flavus*	England, Slovakia	Tend aphids and ↑seeds abundance near mounds	Pontin (1978) and Kovář et al. (2000)
*Lasius neoniger*	USA	↓Lepidoptera (*Agrotis ipsilon*) eggs and larvae	López and Potter (2000)
*Lasius niger*	Belgium, France, Germany	Tend aphids, poor seed disperser, and ↑predator dispersal via chemical cues	Hübner and Völkl (1996), Servigne and Detrain (2008) and Mestre et al. (2014)
*Myrmica americana*	USA	Tend aphids	Bristow (1984)
*Myrmica fracticornis*	USA	Tend aphids	Bristow (1984)
*Myrmica laevinodis*	Germany	Tend aphids	Hübner and Völkl (1996)
*Myrmica lobicornus*	USA	Collect seeds in grasslands	Mittelbach and Gross (1984)
*Myrmica rubra*	Belgium, France, Germany, Russia, USA	↑Herbivores, generalist and predator density, collembola predator, ↓pollination, disperse introduced plant species (in USA), and effective seed disperser	Reznikova and Panteleva (2001), Servigne and Detrain (2008), Sanders and van Veen (2011), Cembrowski et al. (2014) and Prior et al. (2015)
*Myrmica* sp.	USA	Tend membracids with no effect on leaf beetles (*Trirhabda virgata* and *T. borealis*) herbivory	Messina (1981)
*Prenolepis imparis*	USA	Tend membracids with no effect on leaf beetles (*Trirhabda virgata* and *T. borealis*) herbivory	Messina (1981)
*Tapinoma sessile*	USA	Tend aphids	Bristow (1984)
Ants as ecosystem engineers			
*Formica montana*	USA	↑Total N, dissolved organic N, NH_4_^+^ in mound soil	Lane and BassiriRad (2005)
*Formica podzolica*	USA	↑K, PO_4_^−^, Mg, Na, and soil mounding (creating hummocks)	Lesica and Kannowski (1998)
*Formica canadensis*	USA	↓Fe, Zn, Cu, Mn, and plant diversity on mounds	Culver and Beattie (1983)
*Formica fusca*	USA	↑Soil porosity, P, K, Ca, Mg, and pH in mound soil	Levan and Stone (1983)
*Formica obscuripes*	USA	↓Plant species richness, diversity, and abundance with ↑distance from nests	Beattie and Culver (1977)
*Formica rufibarbis*	Germany	↑Na, K, and pH, ↓P, N, and moisture in mound soil, and ↓plant species richness and cover on ant mounds	Dean et al. (1997)
*Formica subsericea*	USA	↑Soil porosity which may extend up to 1 m beyond nest boundaries, ↑organic carbon in mound soil surface, and ↓soil bulk density	Drager et al. (2016)
*Lasius niger*	Germany	Changes in soil properties ↓decomposer density	Sanders and van Veen (2011)
*Lasius claviger*	USA	↑Total N, dissolved organic N, NH_4_^+^ in mound soil	Lane and BassiriRad (2005)
*Lasius alienus*	Germany	↑Na, K, and pH, ↓P, N and moisture in mound soil, and ↓plant species richness and cover on ant mounds	Dean et al. (1997)
*Lasius flavus*	Czech Republic, England, Germany, Slovakia	↑Na, K, P, pH, microbial biomass, organic content, and N-availability in mound soil, ↓P, N, Total C, total N, Ca, Mg, mound soil moisture, and bulk density, ↓plant species richness and cover on ant mounds, and ↑root arbuscular mycorrhizal colonization of grasses through abiotic changes in soil	King (1977a, b), Dean et al. (1997), Dauber et al. (2001), Dostál et al. (2005), Dostál (2007) and Dauber et al. (2008)
*Lasius niger*	Czech Republic, Germany, Slovakia	↑Microbial biomass, organic content, N-availability, C, Ca, pH, and microbial functional diversity in mound soil	Dauber et al. (2001), Dauber and Wolters (2000) and Frouz et al. (2003)
*Myrmica rubra*	Belgium, France, Germany	Changes in soil properties ↓decomposer density	Servigne and Detrain (2008) and Sanders and van Veen (2011)
*Myrmica scabrindis*	Germany	↑Microbial biomass, organic content, N-availability, and microbial functional diversity in mound soil	Dauber et al. (2001) and Dauber and Wolters (2000)

### Ants and herbivores

The impact of ants on herbivores is well documented, with extraordinary examples of ants forming food-for-protection mutualisms (Styrsky and Eubanks 2007; Rosumek et al. 2009). In temperate grasslands, common ant species such as *Lasius* spp. are known to protect aphids and move them between plants (Way 1963; Pontin 1978). Honeydew producing insects can further benefit from ant tending as the result of increased survivorship (e.g., reduced predation), decreased development time, or increased growth (Way 1963; Bristow 1984) (Fig. 2). For example, *Myrmica* spp. tended membracide colonies produce approximately five times more adults, are more likely to survive longer, and are generally larger than adults not tended by ants (Bristow 1984). In turn, by tending honeydew-producing insects, ants maintain a high quality resource within their foraging range that is important for fueling adult workers, and in the case of some *Lasius* spp., that may serve as a significant portion of their protein diet (Pontin 1978). Readily accessible carbohydrate resources are important determining factors regulating colony growth, colony establishment, and worker body size (Wills et al. 2015).

Ant activity associated with tending honeydew-producing insects impacts the presence of other insect herbivores (Messina 1981; Ando and Ohgushi 2008). Ants can reduce herbivore populations by disrupting herbivore feeding (Messina 1981), or by removing them completely (Ando and Ohgushi 2008). Regardless of the presence of honeydew-producing insects, ants are known to contribute to the control of lepidopteran herbivores in grasslands (Sanders and Platner 2007). Ants are often cited as potential and observed predators of lepidopteran eggs and larvae (Sanders and Platner 2007), including monarch butterflies (Zalucki and Kitching 1982) and black cutworm (López and Potter 2000). In extreme cases, such as the introduction of an invasive ant species like the raspberry crazy ant (*Nylanderia fulva*), they can be effective at reducing overall arthropod abundance in grasslands (LeBrun et al. 2013).

### Ants and arthropod predators/parasitoids

Ants in grasslands generally reduce predator populations and interfere with their behavior. Ants impact arthropod predator density through direct consumption, competition, and non-consumptive effects (Sanders and Platner 2007; Sanders and van Veen 2011; LeBrun et al. 2013). In grasslands, spiders are most commonly considered an important generalist arthropod predator, influencing a variety of ecosystem services (Schmitz 2008). Ants prey upon spiders heavily in late spring through early summer (Petal et al. 1971), consuming up to several thousand spiders per square meter every growing season (Petal and Breymeyer 1969). Ant foraging can also reduce spider abundance without any direct consumption of spiders, as the residual chemical cues of ant foraging can also limit their abundance by eliciting spider dispersal or avoidance behavior. Mestre et al. (2014), found that *Phylloneta impressa* exposed to ant chemical cues from *Lasius niger*, a common ant species in European grasslands, increases spider silk dispersal by 80% and more than doubles the dispersal of the hunting spider *Xysticus*.

Ants can also impact the presence of other predators and parasitoids within a grassland when they enter into contact with ant species that tend aphids or other honeydew producing insects (Eubanks et al. 2002; Kaplan and Eubanks 2005). Ant tending species will generally reduce densities of parasitoids (Sanders and van Veen 2011), and in the case of the grassland ant *L. niger*, they can catch and kill around 25% of the parasitoids attacking an aphid colony (Hübner and Völkl 1996). Invasive ant species are even more effective predators. In the US, invasive ant species like the red imported fire ant (*Solenopsis invicta*) can reduce the abundance of important beneficial predators such as green lacewing larvae (Neuroptera: Chrysopidae) and lady beetles (Coleoptera: Coccinellidae) by 38 and 50%, respectively, in defense of their aphid colonies (Eubanks et al. 2002). Ant predation of other predators within grasslands can ultimately positively influence the populations of herbivore and decomposer communities (Kaplan and Eubanks 2005), and herbivory (Messina 1981).

### Ants and arthropod decomposers

Ants are known predators of decomposers in grassland communities potentially impacting decomposer communities directly or via non-consumptive mechanisms as with herbivores and predators. There are relatively few examples documenting their impacts on decomposer abundance in temperate grasslands (Sanders et al. 2011), potentially because of the difficulty in observing the impact of predation on below ground macroinvertebrate communities. However, the impact of ants on decomposers has been observed in other temperate systems, where ants directly consume decomposers (Stoker et al. 1995), or indirectly disrupt decomposer access to food resources (Zhao et al. 2014). For example, in an alpine meadow system in China, an aggressive and territorial ant species (*Camponotus herculeanus*) limited the number of coprophagous beetles and flies landing on dung pats, reducing egg laying success (Zhao et al. 2014).

### Ants and pollinators

As with herbivores, predators, and decomposers, ant foraging can influence the flower visitation behavior of pollinators through direct and indirect interactions. Ants impact pollinator populations by reducing nesting success or consuming pollinators (Zammit et al. 2008). They may also disrupt plant–pollinator relationships and adversely affect plant reproduction (Ness 2006; Galen and Geib 2007). For example, the European fire ant (*Myrmica rubra*) is known to modify foraging behavior of bumblebees, where they transferred and removed more pollen in artificial flowers without ants than flowers with ants, and removed more pollen in flowers without ant scent cues than with ant scent cues (Cembrowski et al. 2014). In their study, Cembrowski et al. (2014) observed ants directly attacking bees visiting the artificial flowers and some bees having trouble flying after the interaction. Similarly, the invasive Argentine ants (*Linepithema humile*) are known to reduce pollinator foraging behavior within the temperate zone (Lach 2008; Sidhu and Wilson Rankin 2016). This aggressive invasive species holds the potential to reduce pollinator visitation duration by 50% and pollinator visitation is three times more likely on flowers without scent cues (Sidhu and Wilson Rankin 2016). These invasive ants may be more likely to impact pollination than native species (Lach 2008), and are worth noting because of their global presence in temperate regions.

Ants can also indirectly reduce pollinator activity or reduce pollination services through competition for floral nectar (Galen and Geib 2007), and in some instances pollen (Byk and Del-Claro 2010). Ants are very rarely effective pollinators (Beattie et al. 1985), and are more often responsible for damaging flowers, or robbing flowers of nectar and pollen resources (Galen and Geib 2007; Byk and Del-Claro 2010). Nectar thieves, like the *Formica neorufibarbus* in high altitude meadows, are known to reduce the median seed set of alpine skypilot flowers (*Polemonium viscosum*) by 16–45% because the ants remove the style to obtain nectar at the base of the flower (Galen 1999; Galen and Geib 2007). These ants potentially further disrupt seed-set as potential predators on flowers or reduced pollinator foraging behavior due to a perceived decrease floral resource availability, but little evidence suggests this occurs here (Galen and Geib 2007). However, through floral damage, resource exploitation, and impact on pollinators and pollination services, ants can have a significant indirect effect on plant fitness and flower morphology (Traveset and Richardson 2006; Galen and Geib 2007).

### Ants and seeds

The role and importance of ants as seed harvesters in grasslands is often dependent on climate. In drier regions, seed harvesting ants typically play an important role in seed dispersal (MacMahon et al. 2000). Seed harvesting ant species are considered seed predators that also opportunistically collect arthropods (see MacMahon et al. 2000). As seed predators, these species will generally consume the entire seed but also disperse seeds when they are lost enroute to, or mistakenly discarded at the nest (Hölldobler and Wilson 1990). In more mesic grasslands, ants can serve as seed dispersers (Dostál 2005; Servigne and Detrain 2008), but are often opportunistic, omnivores (Fiedler et al. 2007) that supplement their diet with seeds when available (Beattie 1989; Servigne and Detrain 2008). In contrast to seed harvesting ants, seed dispersers leave the seed intact and are attracted to seeds with an elaiosome, a lipid rich external appendage of the seeds used to provision their larvae. Once elaiosomes are removed, foragers will discard the seeds. This may occur at any point along the path back to the nest so they are, therefore, considered seed dispersers (Hölldobler and Wilson 1990). In moving seeds away from parent plants, ants can minimize density-dependent effects of seedling competition and mortality from seed predators (e.g., rodents). For example, ants can reduce impacts of seed predation by other seed predators (Manzaneda et al. 2005; Ness and Morin 2008), disperse seeds into new areas (Prior et al. 2015), or transport seeds to ideal seed germination and establishment sites (Beattie and Culver 1983; Hanzawa et al. 1988).

The relative amount of seed dispersal provided by ants in grasslands, therefore, depends on ant community composition (Prior et al. 2015), but is also dependent on seed availability (Servigne and Detrain 2008). For example, *Aphaenogaster rudis*, a species found in temperate North America, is considered a keystone seed disperser of herbaceous plants and often responsible for a disproportionate amount of seed dispersal within temperate systems (Ness et al. 2009). The importance of *A. rudis* as a seed disperser is often studied in the context of temperate forests, but this species is also found in grasslands in the northeastern US (Wodika et al. 2014; Menke et al. 2015), and likely plays a similar role. Species identity can also influence dispersal distance. Based on a global data set, species from the subfamily Myrmicinae, such as *A. rudis*, tend to transport seeds over a shorter distance than those from the subfamily Formicinae (Gómez and Espadaler 1998). The keystone seed disperser, *A. rudis*, is known to move seeds on average 0.63 m, while other grassland species, *Formica subsericea* (Formicinae) have been observed dispersing seeds on average of 12 m (Pudlo et al. 1980). Grassland ant species are also known to collect seeds not associated with ant dispersal, acting as seed predators (Mittelbach and Gross 1984) and contributing to their dispersal in grasslands (Escobar-Ramírez et al. 2011). Ultimately, ants as seed consumers can influence plant diversity within a landscape and are important contributors to plant diversity, abundance, and spatial heterogeneity of plant species (Escobar-Ramírez et al. 2011; Prior et al. 2015).

### Ants and plants

As consumers, ants can influence distribution and diversity of plants within a grassland through one or more interaction described above. The strength of these interactions and impact of ants on plants is further dependent on the species involved (Rosumek et al. 2009) (Fig. 2), and the bottom-up differences in plant quality (Pringle et al. 2014). The strength of the positive interaction of ants as plant protectors tends to increase with ant diversity (Rosumek et al. 2009), but can vary based on species-specific preferences in the honeydew-producing insects they tend and protect (Bristow 1984). The quality of plant-derived carbohydrate resources consumed by ants, through honeydew-producing insects, can determine ant foraging behavior (Pringle et al. 2014). Higher quality resources elicit increased ant foraging behavior on plants and confer a greater level of protection from other herbivores (Pringle et al. 2014). For flowering plants, ant foraging for honeydew or other resources can both disrupt and improve seed set (Messina 1981; Cembrowski et al. 2014), further altering the plant communities within a grassland (Dean et al. 1997). These direct and indirect interactions of ants as consumers have the potential to impact almost all plant life stages and are generally positive. Understanding the relationships of ants within grasslands, particularly their effect on decomposers and herbivores, will be important for determining how best to manage grasslands to maximize its plant and insect biodiversity.

## Ant as ecosystem engineers

Nest construction and associated soil movement by ants can have important implications for soil microbial and invertebrate communities, and influence soil properties. While the percent soil surface occupied by nests of a single species is typically small (< 1–11%), the total surface area occupied by multiple species is rarely estimated (Lobry de Bruyn 1999), and is potentially much larger when one considers their impact on soil properties can extend beyond the surface area of nest structure (Drager et al. 2016). Nest structures also create a mosaic within grassland soils, where nutrients are concentrated and conditions for soil biodiversity increased (Boulton and Amberman 2006). In the following section, we will discuss how ants impact soil communities, soil qualities, and how these in turn can influence the structure and diversity of grassland plant communities. More general reviews exploring the roles of ants on soil properties are also available (Cammeraat and Risch 2008; Frouz and Jilková 2008; Del Toro et al. 2012).

### Ants, invertebrates, and microbes

Nest construction activity and the concentration of food resources within ant nests impact soil conditions and alter important belowground communities. Ants often maintain their nests at a constant temperature that is often higher than ambient air temperature (Frouz and Jilková 2008). Under these conditions of higher availability of resources, nutrients, and constant temperatures, nests can serve as diversity hot spots within a grassland. Nests can be homes to commensal and parasitic insect species (Campbell and Crist 2016), including other ant species such as *Solenopsis molesta* that is commonly found nesting within nests of other species, or social parasitic ant species (e.g., *Polyergus* spp.) that can usurp control within nests and force the host species to raise their offspring (Ellison et al. 2012). In Europe, ant colonies are also hosts to charismatic butterfly species, such as the threatened *Maculinea* butterfly (Thomas et al. 2009). This group of large blue butterflies parasitizes several ant species in the genera *Myrmica*, and can specialize on specific *Myrmica* species (Thomas et al. 2009).

Perhaps more importantly for grassland communities, ant nests can harbor a variety of microorganisms (Boulton et al. 2003; Boulton and Amberman 2006). In drier grasslands in the western US, Boulton and Amberman (2006) found that diversity of nematodes, microarthropods, bacteria, and eukaryotes increases within nests of seed harvesting *Messor andrei*. They also found that the increase in diversity is most likely due to the concentration of nutrients (seeds and insects) rather than an increase in soil moisture due to nest building activity. Regardless of the mechanism, the role of ants in grasslands can have important implications for belowground activity and diversity.

Changes to soil microbial biomass and activity within a nest is strongly influenced by species-specific differences in foraging strategy or nest architecture. In central Germany, Dauber and colleagues found that three common grassland ant species play an important part in determining microbial diversity and activity. They found that the nests of *L. niger* and *Myrmica scabrinodis* are areas of high microbial functional diversity relative to soils with no ant activity, while lower microbial diversity in *Lasius flavus* nests suggesting species-specific differences in their impact on soil communities (Dauber and Wolters 2000). Microbial activity has also been found to be greater in *M. scabrinodis* nests relative to soils with no ant activity, while the nests of *L. niger* and *L. flavus* show no increase in microbial activity (Dauber et al. 2001). Despite limited microbial diversity and activity in *L. flavus* nests, their soil modification activities seem to increase root arbuscular mycorrhizal fungi (AMF) colonization of grasses (Dauber et al. 2008). This increased colonization is potentially the result of AMF spore accumulation and root-spore contact, maintenance of ideal soil temperature, or other soil conditions (e.g., AMF diversity, nutrient availability) (Dauber et al. 2008). Such alterations to soil diversity can have important implications on food webs. Ant nest conditions may influence the numbers of important decomposers such as collembola, which can influence soil nutrient availability to plants, and also serve as important food resources for spiders (Sanders and Platner 2007). Managing grasslands to sustain healthy ant communities may support diverse microbial communities and impact grassland resilience to disturbances.

### Ants, pH, and nutrients

Within nest ant activity can alter soil nutrients and pH via three general processes: (1) ants can move, separate, and alter soil material during nest construction; (2) nest construction can indirectly alter the solubility of nutrients through changes of the nest’s environmental conditions; and (3) ant foraging concentrates food resources and waste products within nests (Cammeraat and Risch 2008). The nature of changes to nest soil are dependent on the original soil conditions. In the case of pH for example, ant activity generally neutralizes nest soil (Frouz and Jilková 2008). For example, a common European grassland species, *L. niger*, tends to increase pH in acidic soil and decrease pH in alkaline soils (Frouz et al. 2003).

Ants can also impact the availability of important nutrients in the soil. A variety of ant species from the genera *Lasius*, *Myrmica*, and *Formica* are common in grasslands across the north temperate regions, and their nests tend to harbor a greater concentration of total nitrogen and phosphorous within the soil (Frouz and Jilková 2008). Ant nests also impact the availability of nutrients within grasslands. Dauber et al. (2001) found that three common grassland species (*M. scabrinodis*, *L. niger*, and *L. flavus*) increase the availability of nitrogen in nest soils. In case of *L. niger*, colonies also tend to have greater available phosphorous than the surrounding soils, because shifts in soil pH increases phosphorus availability (Frouz et al. 2003). However, there are also likely to be species-specific differences in the nutrient availability. For example, *M. scabrinodis* nests have higher levels of available nitrogen than surrounding soils, but is 80% lower than the available nitrogen found in nest of *L. niger* and *L. flavus*, which is likely due to differences in nest structure (Dauber et al. 2001). Nests of *M. scabrinodis* tend to have grassy vegetation integrated into the nest that may reduce nitrogen availability (Dauber and Wolters 2000).

Changes in nutrient availability tends to exhibit temporal turnover which may overall enhance niche diversity. In Illinois grasslands, Lane and BassiriRad (2005) found an increase in total N, dissolved organic N, and NH_4_^+^ on soil from nests of *Formica montana* and *Acanthomyops claviger*, relative to soils from the surrounding prairie soil. Nutritional differences between mound and surrounding soils peaks 8 years post-restoration and differences diminish with time (16, 26 years) since restoration, suggesting that because colony mounds lack vegetation, nest mounds lose the added nutrient input from leaves and roots as experienced by surrounding soils (Lane and BassiriRad 2005). By creating diversity in nutrient concentrations, ants play an important role creating resource heterogeneity within a landscape (Dostál 2007) that may be important in helping to establish restored grasslands on nutrient poor soils.

### Ants, bulk density, and water movement

Ant nest building activity within the soil also modifies soil physical structure, which can ultimately impact how water moves through soil. In constructing nests, ants mix soil from different horizons (bioturbation) (Halfen and Hasiotis 2010). The construction of nest chambers and tunnels, mixes soils and increases pore size. Overall ant nest construction activity reduces soil bulk density, with the exception of nests of species in sandy soils (see Cammeraat and Risch 2008). In Illinois, mound building ant species *F. montana* and *A. claviger* can reduce soil bulk density 60% relative to bulk density of nearby soil (Lane and BassiriRad 2005), a pattern also observed for other mound building ant species in grasslands found within the US (Drager et al. 2016) and Europe (Blomqvist et al. 2000; Dostál et al. 2005). In the case of *F. subsericea*, the changes in bulk density can extend laterally beyond the boundaries of the nest mound, through changes to soil properties directly under the mound structure and ant modifications to natural soil pores extending beyond the nest structures (Drager et al. 2016). By altering the soil physical structure, ant nests can in turn impact water infiltration. Because ants impact organic content within their nests, the direction of impact ants have on water infiltration (increase or decrease) is dependent on surrounding soil conditions. An increase in organic matter can increase water repellency at lower soil moistures (see Frouz and Jilková 2008). Therefore, in wet or moist soils ants can reduce water infiltration and increase water infiltration in drier soils. Belowground nests structures can improve soil drainage (Drager et al. 2016), but *Lasius neoniger*, common to grasslands in temperate North America, are effective at preventing water infiltration into nest structures (Wang et al. 1996). In this case, *L. neoniger* tend to close nest openings prior to precipitation events with up to 80% of nest entrances closed with a minimal amount of precipitation (5 mm) (Wang et al. 1996).

### Ants, soil, and plants

Ant activities within soil have important consequences on belowground biodiversity, nutrient content, and physical properties. These changes have important consequences for grassland plant communities because ant mounds serve as small scale, but frequently-encountered soil disturbances (Umbanhowar 1992). Nest mounds often represent islands of high total nitrogen and phosphorous content, neutral pH, and water content different than the surrounding soils (Frouz and Jilková 2008), but generally do not harbor more plant species than the surrounding soils (Culver and Beattie 1983; Dean et al. 1997). Some ant species, including seed harvesting ants, will directly remove or clip plant vegetation in and around their nests, maintaining vegetation free islands surrounding their nests (MacMahon et al. 2000). However, mounds often do maintain small scale patchiness in grasslands (King 1977b) because in north temperate systems mounds often contain relatively less moisture than surrounding soils. Therefore, nests tend to harbor more xeric tolerant plant species (King 1977a), or other species that cannot compete with the dominant plant species (Beattie and Culver 1983; Dean et al. 1997; Dostál 2007). In drier grasslands with seed-harvesting ant species, nests often serve as important islands for plants by increasing soil moisture and nutrient concentration within nests compared to the surrounding soils (MacMahon et al. 2000). Regardless of the type of grassland, in most cases, ant nests are thought to provide nutrient rich sites that hold the potential for increasing seedling survivorship (Beattie and Culver 1983), or improved plant growth (Dean et al. 1997). For example, when radishes were grown in nest soils from mounds of ants in central Germany (species not specified) and non-nest mound soils, those from nest mound soils had approximately two times as much leaf area, root mass, and shoot mass than radishes grown from the surrounding soil (Dean et al. 1997).

## Future research directions

By summarizing current knowledge of the roles of ants in grasslands and identifying those that need further study, we can improve our understanding of how ants contribute to restoration and management of grasslands. Here, we discuss five avenues for future research specific to north temperate grasslands.

### Dominant disturbance-tolerant ant species

Historic and current changes in land-use have dramatically diminished the connectivity and prevalence of grasslands within landscapes, potentially altering ant community assemblage processes and community structures. Species-specific changes to an ant community can alter seed dispersal (Ness et al. 2009), populations of ant specialist nest parasites (Thomas et al. 2009), nest conditions and subsequent changes to soil conditions (Dauber et al. 2001), or many other interactions. Efforts to restore grasslands can help maintain diverse ant communities, but restored grasslands are not always similar to those in remnant grasslands (Német et al. 2016). This is potentially attributable to the lack of connectivity between grasslands, leaving some species unable to enter newly established grasslands (Német et al. 2016), or because dominant species prevent establishment of other species (Moranz et al. 2013). Dominant disturbance-tolerant ant species are often invasive species (LeBrun et al. 2013), and they may also be an important factor influencing populations of native ant species (Moranz et al. 2013). Future research should consider the importance of native ant species that are disturbance-tolerant generalists and explore how they impact the establishment of other grassland ant species. We can use this information when managing grasslands to minimize dominant species from excluding other species, potentially increasing overall ant biodiversity.

### Invasive ant species

Introduced generalist species of ants often displace existing ant species, disrupting their functional roles (Prior et al. 2015), and consuming a variety of native arthropods (LeBrun et al. 2013). The introduced ant species the red imported fire ant (*S. invicta*) can become predominant features in the southern US, and despite their ecosystem disservices, may provide some benefits. They can serve as biocontrol of some plant pests (Styrsky and Eubanks 2007) and may even play a role in improving soil quality (Lafleur et al. 2005). In the cases like *S. invicta*, where control efforts have largely failed and they will likely remain a permanent feature of the landscape, we should start considering their ecosystem services, particularly their role in belowground soil processes. However, this does not suggest we should reduce efforts to document, control, or mitigate the impacts of other invasive ant species such as the Japanese pavement ant (*Tetramorium tsushimae*) and the European fire ant (*M. rubra*) whose introductions are more isolated and relatively recent. Moreover, because the ecological conditions of these species' introduced ranges are so similar to their native ranges, these introduced ant species may have important implications for biotic interactions in north temperate regions. For example, *M. rubra* is known to disperse more seeds of an invasive plant (*Chelidonium majus*) than *A. rudis*, a native a keystone disperser (Prior et al. 2015). When introduced ant species interact with other introduced species, they hold the potential to further alter an already disturbed ecological community through their synergistic interactions (Simberloff and Von Holle 1999). Identifying and understanding the impact of these relationships, particularly between co-evolved species (e.g., ant–plant relationships), will be important as species are moved around the globe.

### Pest suppression

Ants may play an important role in natural control of pest populations within grasslands. For example, ants have been identified as important predators of pest lepidopteran (López and Potter 2000) and coleopteran larvae (Zhao et al. 2014), and may be contributing to pest control in surrounding agricultural fields. In perennial crops such as blueberry, ants have been observed foraging day and night and are quick to recruit and remove sentinel prey items (Grieshop et al. 2012). As social insects, ants in general are at an advantage when food resources are clustered. As a unit, foraging workers can cover more area than an individual organism. Once a resource is located, ants quickly recruit to, retrieve, or defend a resource. Additionally, because of their association with honeydew producing insects and extrafloral nectar, ant foraging on plant surfaces may limit herbivore populations through predation or disturbance (e.g., non-consumptive effects). Even the notorious invasive *S. invicta* can in some cases limits herbivory on cotton (Eubanks 2001). Ants are unlikely to be important predators in traditional annual cropping systems (e.g., corn and soy) due to the soil disturbance of tillage, but in the north temperate region they may serve important roles in perennial crops and create pest population sinks in grassland habitats surrounding crop fields. More work is required to understand their role as natural pest control in grasslands within the agricultural landscape.

### Soil processes

Ants can be abundant members of soil communities, yet little research has been focused on their impact on soil community structure and function. With the advent of new tools and lower costs for next generation sequencing, we can further explore how ant activity and diversity impact soil microbial communities and soil processes. The effect of ants often differs based on soil quality (Frouz and Jilková 2008), and using sequencing tools we can potentially explore how ants impact soil microbial functional diversity across a variety of habitat types to build a broader understanding of the role of ants in belowground processes. Moreover, because the impact of ants on soil communities can be species-specific (Dauber and Wolters 2000), we can also start identifying particular species or taxonomic groups that play a larger role in influencing soil microbial communities. One potential candidate is *L. neoniger*, because it is common and numerically dominant in restored grasslands (Wodika et al. 2014). This species is also known to influence soil turnover and alter physical properties of the soil (Wang et al. 1995) and, therefore, may exert a disproportional influence on soil processes in restored or disturbed grasslands.

### Climate change

In each of the future research directions listed above, climate change represents an additional factor which may alter the function of ants in grasslands. Ant species physiological limits are important factors determining their species-specific responses to climate change, and useful to helping forecast their presence and responses to future climate conditions (Diamond et al. 2012). In general, because north temperate ant species are further from their critical thermal maximum they are more resilient to changes in climate than tropical species (Diamond et al. 2012, 2013). Furthermore, increases in air temperature can impact species ranges, with the potential for cold-intolerant invasive species (e.g., *S. invicta*) to move into previously uninvaded ranges (Morrison et al. 2004). However, ants display incredibly plastic responses to cope with changes in temperature. For example in hot, dry climates smaller bodied *Cataglyphis velox*, workers forage during the cooler morning hours and larger workers forager during the heat of the day (Cerdá and Retana 2000). Within a habitat, ants can also adjust a nest’s location and architecture to improve nest thermoregulation in response to changes in temperature (Jones and Oldroyd 2006). Overall, north temperate ants as a whole are likely able to be resilient to moderate changes in temperature (Diamond et al. 2017).

However, this resilience does not exclude behavioral shifts that may disrupt the role of ants, the stability of ant communities and the role of ants as consumers and ecosystem engineers. Niches of grassland ant communities are portioned in part by temperature and humidity preferences (Albrecht and Gotelli 2001). Shifts in climate may destabilize ant communities, limit foraging periods, and disrupt their important biotic interactions with other grassland species. Additionally, changes in climate may also shift the phenology of plants dependent on ant seed dispersal, causing an uncoupling of plant fruiting and peak foraging activity of keystone dispersers (e.g., *A. rudis*). Shifts in nest construction activities can also alter the soil microbial community, soil turnover, and soil porosity that ultimately impacts the role of ants as ecosystem engineers. Finally, changes in climate may impact colony investment in timing and rate of colony growth and subsequently their ecosystem services. This may occur through changes in proximate, temperature cues in colony development, changes to food availability or foraging success, or larval development.

Future research should consider the implications and consequences of climate change on ants and the subsequent changes to their interactions listed above. For example, predictive models have been developed to forecast the effects of climate change on ant communities, they lack important information regarding impact on colony demographics beyond worker survival (Diamond et al. 2013). Quantifying and incorporating the impacts of climate change on species interactions into predictive models is daunting. To overcome this, a primary focus should be to identify keystone ant species and their key community interactions to move closer to developing a better understanding of climate change impacts on temperate grassland systems. Future work that includes how climate change impacts foraging, phenology (colony and food resources), nest construction, and colony growth will be valuable when developing models that generalize how climate change may impact ant communities (Fitzpatrick et al. 2011).

### Grassland management

Due to the continuing loss of native grasslands, land managers will be integral in maintaining ant communities and their roles within grasslands. Ant communities in grasslands respond differently to conservation and management efforts than plant communities (Englisch et al. 2005). Land managers will need to be aware of how ants respond to different management strategies. For example, ant diversity appears to be locally determined by the structural properties of the vegetation that in turn influences soil temperature (Dauber and Wolters 2005). Soil quality and humidity also influence colony establishment within grasslands (Dauber et al. 2005). Managers can reduce plant litter, improving light penetration to the soil surface, and provide soil conditions can increase ant abundance and diversity by utilizing fire, grazing, and mowing regimes in grassland management plans (Moranz et al. 2013). Future work on how to mitigate the effects of fragmentation and habitat constriction on ant communities will be important in determining how land managers can conserve ant diversity. For example, determining, how best to add and link grasslands will be important for conserving ant species, and maintaining grassland landscape diversity.

Future work should continue to identify ant species that play a disproportionally important role in grassland communities and describe the breadth of their influence. As we have identified in this review, these ant species typically are generalists and numerically dominant species that play a role in multiple ecosystem processes (e.g., *L. neoniger*, *L. niger*, *L. flavuus*, and *A. rudis*). These and other key species often require different habitat conditions (Thomas et al. 2009). Future work should first identify key species to their region, their habitat requirements, and then adjust management practices to maintain the desired functions associated with these species within the landscape (Thomas et al. 2009). Currently, relatively more work has explored how different management techniques impact ant communities in European grasslands (Dahms et al. 2005), but relatively less for temperate North America (Nemec 2014, but see Moranz et al. 2013). Moreover, because of the variation in grasslands across North America (e.g., short-grass, mixed, and tallgrass prairie) additional work is needed to develop best management practices for maintaining ant communities for different regional type grasslands.

Generally, it appears most dominant, generalist species are effective contributors to multiple processes. Thus, maintaining their abundance should require relatively little changes to current management strategies. However, land managers will also need to retain other ant species as this will improve performance and resilience of ecosystem services. To do so, land managers can manage individual parcels to create and maintain high quality local habitats (e.g., through fire, grazing) that disrupt competitively dominant ant species (Moranz et al. 2013). They should also avoid major soil disturbances during the peak periods of colony founding (region dependent) to improve their impact on soil processes. Ultimately, by developing regional management strategies to maximize connectivity, and patch diversity in age, disturbance regime, and size at the landscape scale, managers will maximize the beneficial services ants provide.

## Conclusion

Ants are among the most diverse and successful insects in terrestrial ecosystems. Their abundance, diversity, and biomass make them important consumers and ecosystems engineers (Del Toro et al. 2012). Overall ants appear to play a positive role in grasslands. Previously published meta-analyses suggest the roles of ants as consumers is generally positive for plants, serving to protect plants from herbivory (Styrsky and Eubanks 2007; Rosumek et al. 2009). Moreover, this and prior reviews of their role ants play as ecosystem engineers in a variety of systems have found that ants generally improve soil conditions supporting greater plant diversity (Del Toro et al. 2012; Nemec 2014). Ants can also serve as key contributors to a variety of ecosystem processes that help maintain a more resilient ecosystem in the face of major disturbance and habitat loss. Future work should identify key members of north temperate grassland communities, such as dominant generalist ant species and invasive species, and their impact on community assembly and key ecosystem processes. Moreover, additional work is needed to fully understand the role of ants in natural control of pest populations and the regulation of soil processes. Finally, understanding how ant communities and their associated interactions are impacted by climate change and management will be essential for mitigating the loss of biodiversity and ecosystem services in an ever changing world. By identifying key species and strengthening our understanding of their ecological function within grasslands and other temperate systems, we can develop monitoring programs that can quickly assess the effects of changes to land management or land use (Underwood and Fisher 2006; Nemec 2014). This will bolster efforts to restore and manage grasslands for recreation, biofuel feedstocks, aesthetics, ecosystem services, or biodiversity. Ultimately, by maintaining diverse and resilient grasslands within the landscape, we can maximize ecosystem services.
